# Fluorescent polymer films based on photo-induced electron transfer for visualizing water†

**DOI:** 10.1039/d2ra03894c

**Published:** 2022-09-09

**Authors:** Saori Miho, Keiichi Imato, Yousuke Ooyama

**Affiliations:** Applied Chemistry Program, Graduate School of Advanced Science and Engineering, Hiroshima University 1-4-1 Kagamiyama Higashi-Hiroshima 739-8527 Japan yooyama@hiroshima-u.ac.jp

## Abstract

As fluorescent materials for visualization, detection, and quantification of a trace amount of water, we have designed and developed a PET (photo-induced electron transfer)-type fluorescent monomer SM-2 composed of methyl methacrylate-substituted anthracene fluorophore-(aminomethyl)-4-cyanophenylboronic acid pinacol ester (AminoMeCNPhenylBPin) and achieved preparation of a copolymer poly(SM-2-*co*-MMA) composed of SM-2 and methyl methacrylate (MMA). Both SM-2 and poly(SM-2-*co*-MMA) exhibited enhancement of the fluorescence emission with the increase in water content in various solvents (less polar, polar, protic, and aprotic solvents) due to the formation of the PET inactive (fluorescent) species SM-2a and poly(SM-2-*co*-MMA)a, respectively, by the interaction with water molecules. The detection limit (DL) of poly(SM-2-*co*-MMA) for water in the low water content region below 1.0 wt% in acetonitrile was 0.066 wt%, indicating that poly(SM-2-*co*-MMA) can act as a PET-type fluorescent polymeric sensor for a trace amount of water in solvents, although it was inferior to that (0.009 wt%) of SM-2. It was found that spin-coated poly(SM-2-*co*-MMA) films as well as 15 wt% SM-2-doped polymethyl methacrylate (PMMA) films produced a satisfactory reversible fluorescence off–on switching between the PET active state under a drying process and the PET inactive state upon exposure to moisture, which is demonstrated by the fact that the both the films are similar in hydrophilicity to each other from the measurement of the water contact angles on the polymer film surface. Herein we propose that PET-type fluorescent polymer films based on a fluorescence enhancement system are one of the most promising and convenient functional dye materials for visualizing moisture and water droplets.

## Abstract 2

Photo-induced electron transfer (PET)-type fluorescent polymer films based on a fluorescence enhancement system have been prepared as one of the most promising and convenient functional dye materials for visualizing moisture and water droplets.

## Graphical abstract

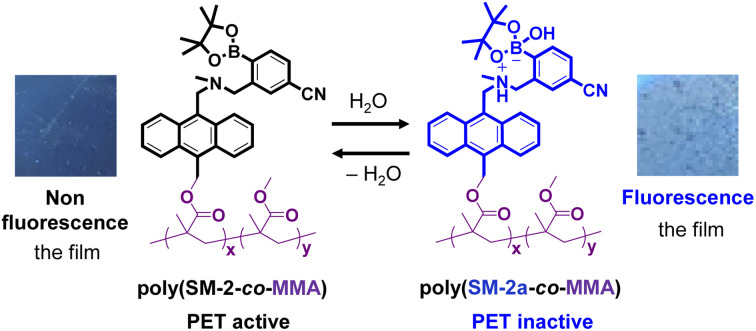

## Introduction

**Fig. 1:**
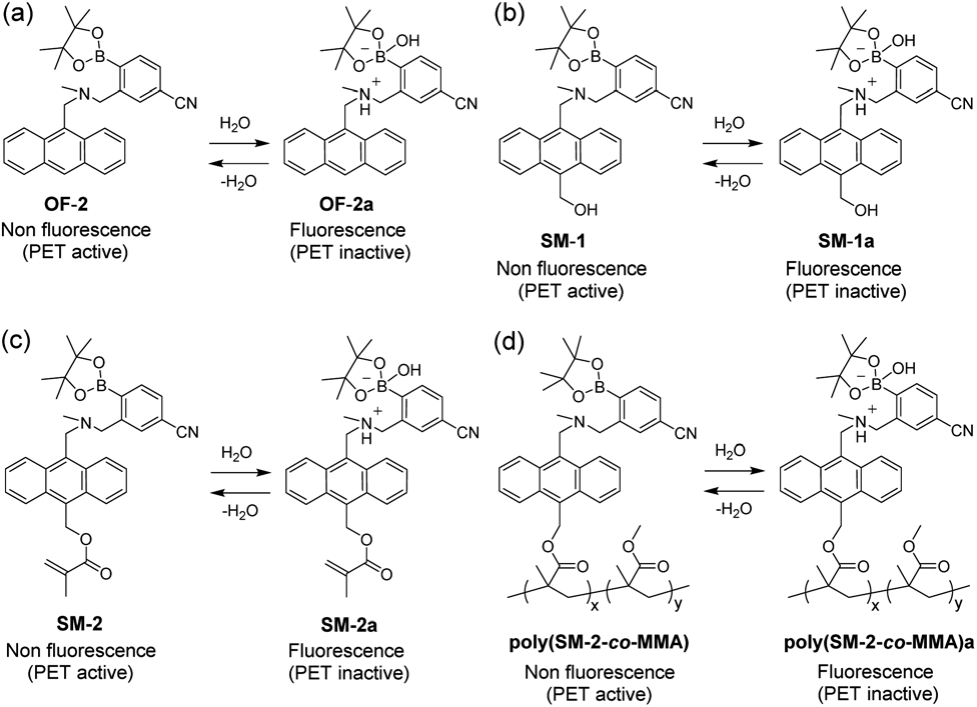
Mechanisms of PET-type fluorescent sensors (a) OF-2, (b) SM-1 (previous work), (c) SM-2, and (d) poly(SM-2-*co*-MMA) (this work) for detection of water in organic solvents and films.

In recent years, concern has been raised about the development of fluorescent sensors and their functional materials such as polymer films and sensor-immobilized membranes for visualizing water in solutions, solids, and gas or on material surfaces, from the viewpoint of their potential applications to environmental and quality control monitoring systems and industry, as well as fundamental study in photochemistry, analytical chemistry, and photophysics.^1–23^ Several investigations have been conducted on the design and synthesis of organic fluorescent sensors and polymers for the detection of water based on ICT (intramolecular charge transfer),^24–34^ ESIPT (excited state intramolecular proton transfer),^35–38^ PET (photo-induced electron transfer),^39–46^ or solvatochromism^47–52^ and the elucidation of the optical sensing properties based on changes in wavelength, intensity, and lifetime of fluorescence emission depending on the water content. It was demonstrated that most of ICT- and ESIP-type fluorescent sensors and fluorescent conjugated polymers exhibited attenuation of the fluorescence emission, that is, fluorescence quenching (turn-off) systems with the increase in water content in solvents, and were suitable for the detection and quantification of a trace amount of water (below 1–10 wt% in almost every case) in solvents. However, one can see that the fluorescence quenching (turn-off) systems make it difficult to visually confirm the presence of water in samples and on material surfaces. On the other hand, the PET-type fluorescent sensors are based on a fluorescence enhancement (turn-on) system and showed the increase in the fluorescence intensity with the increase in water content in solvents, so that it allowed us to visually confirm the presence of water in samples and on material surfaces. Thus, we have focused on the design and development of PET-type fluorescent sensors for the detection and quantification of water and the preparation of their functional materials for visualizing water. In our continuous work for the improvement of the sensitivity and accuracy of PET-type fluorescent sensors for water during the past decade, we have demonstrated that anthracene-(aminomethyl)-4-cyanophenylboronic acid pinacol ester (AminoMeCNPhenylBPin) OF-2 and its derivative SM-1 having a hydroxymethyl group on the anthracene skeleton were highly sensitive PET-type fluorescence sensors for the detection and quantification of a trace amount of water in polar, less polar, protic, and aprotic solvents (Fig. 1a and b).^23,42^ In each sensor, the PET takes place from the nitrogen atom of the amino moiety to the photoexcited anthracene fluorophore in the absence of water, leading to quenching of the fluorescence. When water was added to the solution of OF-2 or SM-1, the nitrogen atom of the amino moiety was protonated or strongly interacted with water molecules to form the PET inactive (fluorescent) species OF-2a or SM-1a, and as a result, a drastic enhancement of the fluorescence emission was observed due to the suppression of PET. Indeed, the detection limits (DLs) and quantitation limits (QLs) of OF-2 and SM-1 for water in acetonitrile were, respectively, 0.009 wt% and 0.026 wt% and 0.004 wt% and 0.013 wt%, which were equivalent or superior to those of the fluorescence quenching systems (turn-off) based on the reported ICT-type^24–34^ and ESIPT-type^35–38^ fluorescent sensors. Thus, the PET method based on the fluorescence enhancement (turn-on) system makes it possible to visualize, detect, and determine a trace amount of water in solvents.

Meanwhile, under the Coronavirus Disease 2019 (COVID-19) situation, face shields made of polyester or polycarbonate films and partitions made of acrylic resin are one of convenience and commercially available protective goods for reducing the risk of droplet infection. Therefore, if we can visually confirm the presence of droplets containing infectious viruses on the face shields and partitions, this allows us to accurately remove the viruses by wiping away the droplets. However, the virus-containing droplets are generally 5 μm or more which is too small for our eyes to see. Nevertheless, because over 90% of the droplets is composed of water, functional materials as well as techniques and methods capable of visualizing water are undoubtedly useful for detecting the virus-containing droplets. In our previous work, for this purpose, we have achieved the preparation of various types of fluorescent polymer films (polystyrene, poly(4-vinylphenol), polyvinyl alcohol, and polyethylene glycol) doped with the PET-type fluorescent sensor OF-2 or SM-1.^22,23^ It was found that the OF-2- or SM-1-doped polymer films exhibited a reversible switching of the fluorescent color between feeble green excimer emission in the PET active state under a drying process and intense blue monomer emission in the PET inactive state upon exposure to moisture or water droplets. Our previous work is the first to achieve the preparation of PET-type fluorescent sensor-doped polymer films for water, while ICT-type^32–34^ or ESIPT-type^37,38^ fluorescent sensor-doped polymer films and fluorescent conjugated polymers^18–21,53,54^ for water based on a fluorescence quenching (turn-off) system have been reported. However, the reversibility of the fluorescence intensity of OF-2- or SM-1-doped polymer films between the excimer and monomer emissions in the dry–wet process were not fully satisfactory for the practical use for the visualization and detection of water on materials surfaces, due to destruction of the films during the dry–wet process.

Thus, in this work, to improve the reversibility of the fluorescence intensity of PET-type fluorescent polymer films by giving strong durability during the dry–wet process, we have designed and developed a PET-type fluorescent monomer SM-2 having a methyl methacrylate group on the anthracene skeleton as a derivative of SM-1 and achieved preparation of a copolymer poly(SM-2-*co*-MMA) composed of SM-2 and methyl methacrylate (MMA) (Fig. 1c and d). It was found that spin-coated poly(SM-2-*co*-MMA) films as well as SM-2-doped polymethyl methacrylate (PMMA) films produced a satisfactory reversible fluorescence off–on switching between the PET active state under a drying process and the PET inactive state upon exposure to moisture, which is demonstrated by the fact that the both the films are similar in hydrophilicity to each other from the measurement of the water contact angles on the polymer film surface. Herein we propose that PET-type fluorescent polymer films based on a fluorescence enhancement system are one of the most promising and convenient functional dye materials for visualizing moisture and water droplets.

## Results and discussion

**Scheme 1:**
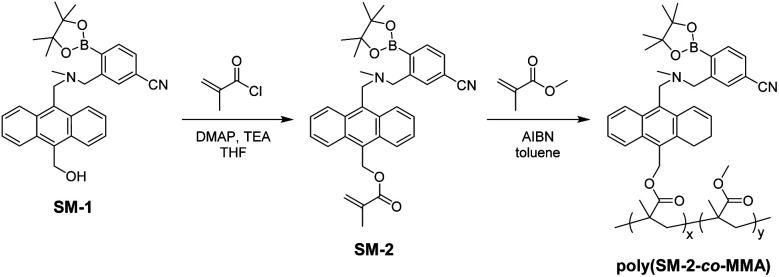
Synthesis of SM-2 and poly(SM-2-*co*-MMA).

The PET-type fluorescent monomer SM-2 was prepared by the reaction of SM-1^23^ with methacryloyl chloride (Scheme 1). Then, polymerization was carried out by a ratio of SM-2 and MMA of 1 : 20 using 2,2′-azobis(isobutyronitrile) (AIBN)^55^ as a free radical initiator to give poly(SM-2-*co*-MMA) as a white solid (*M*_*n*_ = 18 900, *M*_w_/*M*_*n*_ = 2.08, 17% yield). As the result, the ^1^H NMR spectrum indicated that the molar ratio (*y*/*x*) of MMA unit (*y*) and SM-2 unit (*x*) and the weight percentage (wt%) of SM-2 in the obtained poly(SM-2-*co*-MMA) were determined to be *ca.* 40 and *ca.* 15 wt%, respectively.

**Fig. 2:**
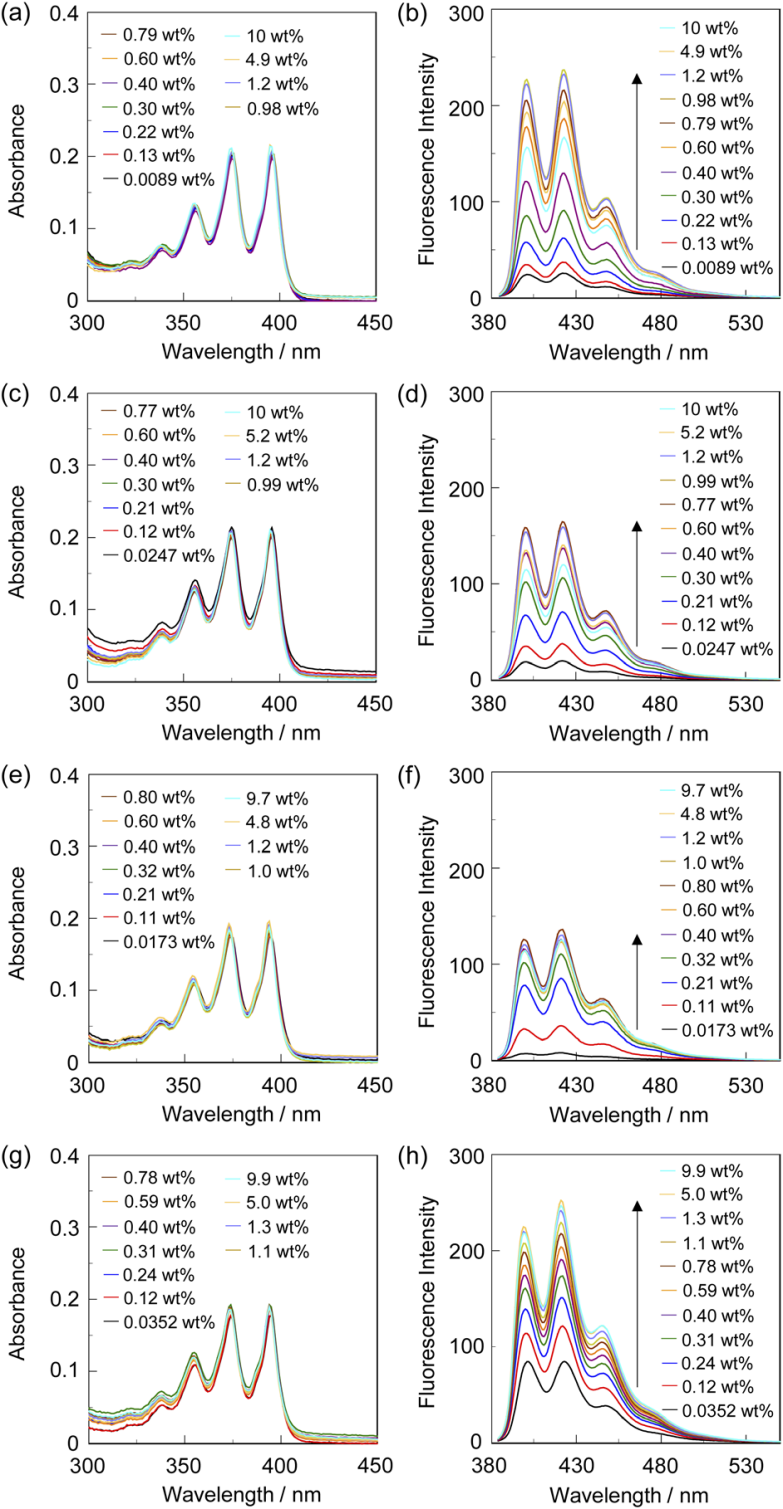
(a) Photoabsorption and (b) fluorescence spectra (*λ*^ex^ = 375 nm) of SM-2 (2.0 × 10^−5^ M) in 1,4-dioxane containing water (0.0089–10 wt%). (c) Photoabsorption and (d) fluorescence spectra (*λ*^ex^ = 375 nm) of SM-2 (2.0 × 10^−5^ M) in THF containing water (0.0247–10 wt%). (e) Photoabsorption and (f) fluorescence spectra (*λ*^ex^ = 374 nm) of SM-2 (2.0 × 10^−5^ M) in acetonitrile containing water (0.0173–9.7 wt%). (g) Photoabsorption and (h) fluorescence spectra (*λ*^ex^ = 374 nm) of SM-2 (2.0 × 10^−5^ M) in ethanol containing water (0.0352–9.9 wt%).

**Fig. 3:**
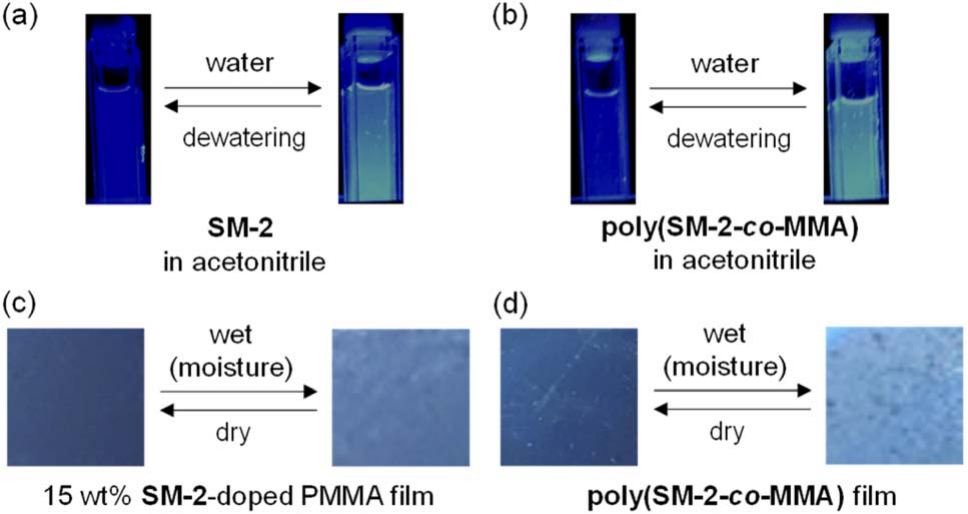
Photographs (under 365 nm irradiation) of acetonitrile solutions of (a) SM-2 and (b) poly(SM-2-*co*-MMA) before and after addition of water and (c) 15 wt% SM-2-doped PMMA film and (d) poly(SM-2-*co*-MMA) film before and after exposure to moisture.

**Fig. 4:**
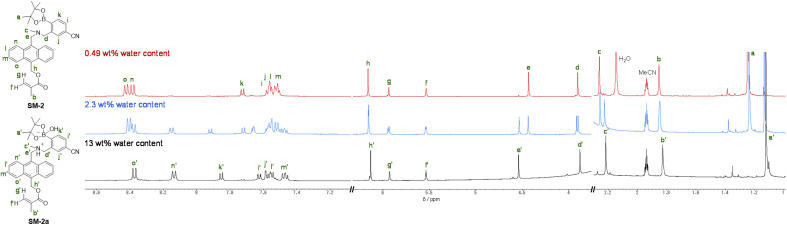
^1^H NMR spectra of SM-2 (2.0 × 10^−2^ M) in acetonitrile-*d*_3_ with 0.49 wt%, 2.3 wt%, and 13 wt% water content.

The optical sensing ability of the PET-type fluorescent monomer SM-2 for water in solvents was investigated by photoabsorption and fluorescence spectral measurements in 1,4-dioxane and THF as less polar solvents, acetonitrile as a polar solvent, and ethanol as a protic solvent containing various concentrations of water (in the water content region below 10 wt%) (Fig. 2). As with the cases of OF-2^42^ and SM-1,^23^SM-2 in all the four solvents showed a vibronically-structured photoabsorption band in the range of 300 nm to 400 nm originating from the anthracene skeleton and did not undergo appreciable changes in the absorbance and shape upon the addition of water to the solutions (Fig. 2a, c, e and g). For the corresponding fluorescence spectra, SM-2 in the absolute solvents exhibited a feeble and vibronically-structured fluorescence band with a fluorescence maximum wavelength (*λ*^fl^_max_) at around 420 nm in the range of 400 nm to 500 nm, which is attributed to the monomer emission originating from the anthracene fluorophore in the PET active state (Fig. 2b, d, f and h). On the other hand, in the low water content region below 1.0 wt%, the fluorescence band increased in the intensity with the increase in the water content in the solution, which is attributed to the formation of the PET inactive (fluorescent) species SM-2a by the addition of a water molecule, as with the cases of OF-2^42^ and SM-1^23^ (Fig. 1a–c). As shown in Fig. 3a, the acetonitrile solution of SM-2 without the addition of water did not show visual fluorescence emission but exhibited the blue fluorescence emission originating from the anthracene fluorophore upon the addition of water. In fact, to confirm the formation of the PET inactive species SM-2a by the interaction with a water molecule, we performed ^1^H NMR spectral measurements of SM-2 with and without the addition of water in the acetonitrile-*d*_3_ solution (2.0 × 10^−2^ M) (Fig. 4). The ^1^H NMR spectrum of the SM-2 solution (water content of 0.49 wt%) without the addition of water showed an obvious signal that can be assigned to a single chemical species with the SM-2 structure. On the other hand, some additional signals appeared in both the aliphatic and aromatic regions in the ^1^H NMR spectrum of the SM-2 solution with water content of 2.3 wt%, compared to that of the solution without the addition of water, indicating the existence of other chemical species as well as SM-2. Moreover, for the ^1^H NMR spectrum of the SM-2 solution with water content of 13 wt%, the chemical shifts of the methyl protons H_a_ of boronic acid pinacol ester, the aminomethyl protons H_c_, and the aromatic protons H_n_ and H_o_ of the anthracene skeleton showed considerably upfield shifts, while those of the methylene protons H_e_ next to the anthracene skeleton and the aromatic protons H_i_ and H_k_ of the phenyl group showed considerably downfield shifts. Consequently, the fact strongly indicates that the PET inactive species SM-2a interacted with water molecules occurred upon the addition of water to the SM-2 solution (Fig. 1c), as with the cases of OF-2^42^ and SM-1.^23^

**Fig. 5:**
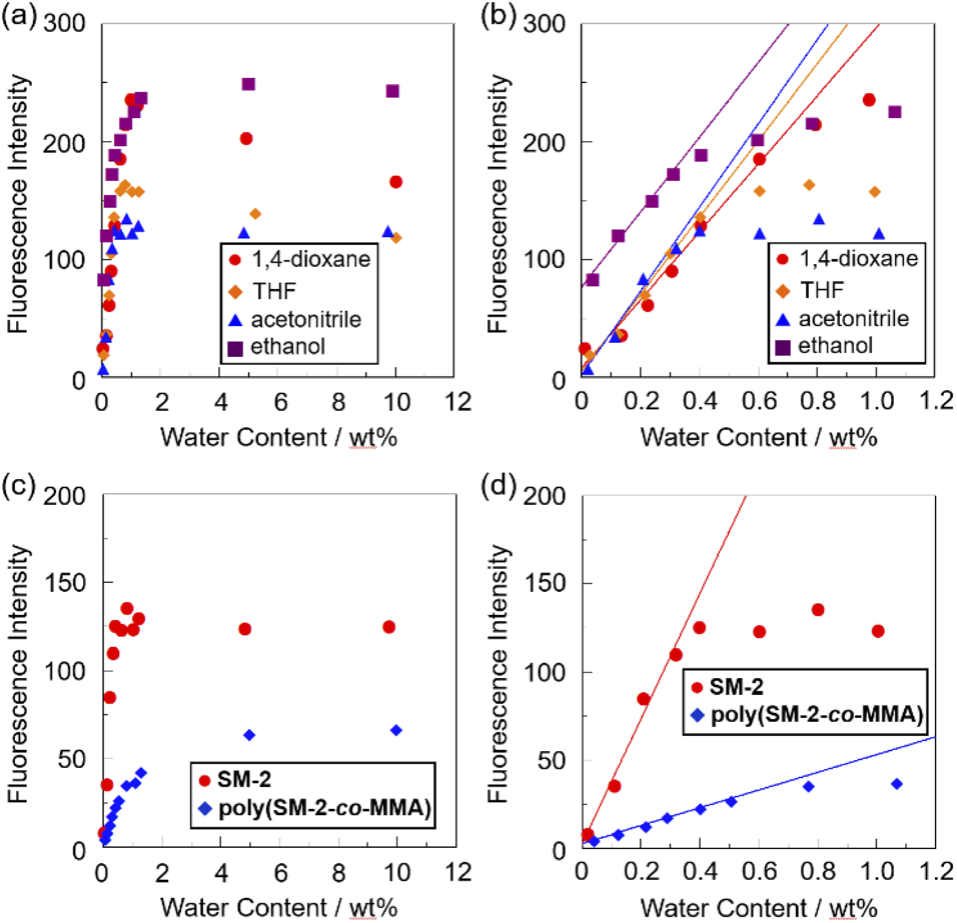
Fluorescence peak intensity at around 420 nm of SM-2 (*λ*^ex^ = 374 or 375 nm) as a function of water content below (a) 10 wt% and (b) 1.1 wt% in 1,4-dioxane, THF, acetonitrile, and ethanol. Fluorescence peak intensity at around 420 nm of SM-2 (*λ*^ex^ = 374 nm) and poly(SM-2-*co*-MMA) (*λ*^ex^ = 375 nm) as a function of water content below (c) 10 wt% and (d) 1.1 wt% in acetonitrile.

The sensitivity and accuracy of SM-2 for the detection of water in solvents were evaluated by the changes in the fluorescence peak intensity at around 420 nm and the plots against the water fraction in solvents (Fig. 5). The plots for SM-2 demonstrated that the fluorescence peak intensity increased linearly as a function of the water content in the low water content region below 1.0 wt% in all four solvents (Fig. 5a), while the fluorescence intensity leveled off when the water content reached 1.0 wt% as with the cases of OF-2^42^ and SM-1.^23^ The results of the plots for SM-2 are as follows:

1 1,4-Dioxane: *F* = 288.6[H_2_O] + 7.99 (*R*^2^ = 0.974, [H_2_O] = 0.0089–0.60 wt%)

2 THF: *F* = 326.0[H_2_O] + 5.32 (*R*^2^ = 0.986, [H_2_O] = 0.0247–0.40 wt%)

3 Acetonitrile: *F* = 355.5[H_2_O] + 2.10 (*R*^2^ = 0.977, [H_2_O] = 0.0173–0.32 wt%)

4 Ethanol: *F* = 317.6[H_2_O] + 76.7 (*R*^2^ = 0.988, [H_2_O] = 0.0352–0.31 wt%)

The correlation coefficient (*R*^2^) values for the calibration curves of SM-2 were 0.974–0.988, which indicates good linearity. A linear change in fluorescence intensity as a function of water content is one of the factors required for the practical use of a fluorescence sensor for water.^39–43^ The intercept values (2.1–76.7) demonstrated that the plots for 1,4-dioxane, THF, and acetonitrile fit straight lines passing through almost the origin, which also indicates the fluorescence enhancement due to the formation of the PET inactive species SM-2a with the increase in the water content. Meanwhile, it is considered that the enhanced fluorescence of SM-2 in absolute ethanol is attributed to the suppression of PET by the hydrogen bonding between the hydroxyl group of ethanol and the amino moiety of SM-2, as with the cases of OF-2^42^ and SM-1.^23^ It is worth mentioning here that there was a little difference in the *m*_s_ values (288–355) for SM-2 between the four solvents, while the *m*_s_ values for SM-2 were equivalent to those for OF-2 but smaller than those for SM-1 (Table 1). The large *m*_s_ values for SM-1 relative to SM-2 and OF-2 can be attributed to the fact that the fluorescence emission property was improved by the introduction of a hydroxymethyl group to the anthracene fluorophore. Actually, fluorescence quantum yields (*Φ*_fl_) of OF-2, SM-1, and SM-2 in absolute acetonitrile were below 2%, but in acetonitrile with 1.0 wt% water content, the *Φ*_fl_ (20%) of SM-1 was higher than those (13% and 12%, respectively) of OF-2 and SM-2. The DLs and QLs of SM-2 for water in the solvents were determined based on the following equations: DL = 3.3*σ*/*m*_s_ and QL = 10*σ*/*m*_s_, where *σ* is the standard deviation of blank sample and *m*_s_ is the slope of a calibration curve obtained from the plot of the fluorescence peak intensity at around 420 nm the water fraction in the low water content region below 1.0 wt% (Fig. 5b). The DLs and QLs of SM-2 for water were, respectively, 0.011 and 0.035 wt% in 1,4-dioxane, 0.01 and 0.03 wt% in THF, 0.009 and 0.028 wt% in acetonitrile, and 0.01 and 0.032 wt% in ethanol, which were equivalent to those of OF-2 but inferior to those of SM-1. Consequently, it was found that methyl methacrylate-substituted anthracene-AminoMeCNPhenylBPin SM-2 can act as a PET-type fluorescent sensor for the detection and quantification of a trace amount of water in polar, less polar, protic, and aprotic solvents, as with the reported PET-type fluorescent sensors for water including OF-2 and SM-1.

**Table 1:** DLs and QLs of OF-2, SM-1, SM-2, and poly(SM-2-*co*-MMA) for water in various organic solvents

Sensor	Solvent	*m*_s_ a	DL^b^	QL^c^
OF-2^42^	1,4-Dioxane	334	0.01 wt%	0.03 wt%
	THF	390	0.008 wt%	0.026 wt%
	Acetonitrile	382	0.009 wt%	0.026 wt%
	Ethanol	362	0.009 wt%	0.027 wt%
SM-1^23^	1,4-Dioxane	564	0.006 wt%	0.018 wt%
	THF	819	0.004 wt%	0.012 wt%
	Acetonitrile	753	0.004 wt%	0.013 wt%
	Ethanol	484	0.007 wt%	0.021 wt%
SM-2	1,4-Dioxane	288	0.011 wt%	0.035 wt%
	THF	326	0.01 wt%	0.03 wt%
	Acetonitrile	355	0.009 wt%	0.028 wt%
	Ethanol	317	0.01 wt%	0.032 wt%
poly(SM-2-*co*-MMA)	Acetonitrile	50	0.066 wt%	0.2 wt%

a Slope of calibration curve.

b Detection limit (DL) and quantitation limit (QL) of sensor for water.

c Detection limit (DL) and quantitation limit (QL) of sensor for water.

**Fig. 6:**
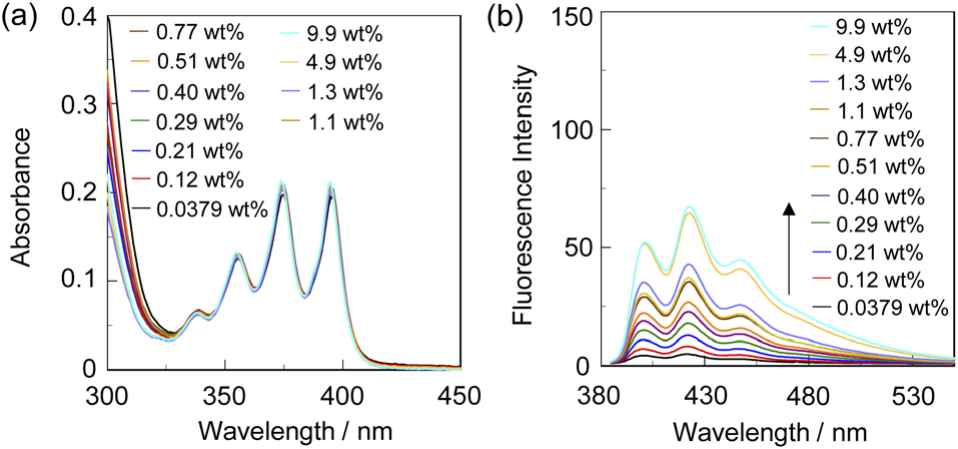
(a) Photoabsorption and (b) fluorescence spectra (*λ*^ex^ = 375 nm) of poly(SM-2-*co*-MMA) in acetonitrile containing water (0.0379–9.9 wt%). The concentration of the solutions was adjusted so that the absorbance at 375 nm was *ca.* 0.2, that is, the concentration of SM-2 unit in poly(SM-2-*co*-MMA) was *ca.* 2.0 × 10^−5^ M, which was the same as the case of SM-2 in Fig. 2.

To investigate the optical sensing ability of the copolymer poly(SM-2-*co*-MMA) for water, photoabsorption and fluorescence spectra of poly(SM-2-*co*-MMA) were measured in acetonitrile containing various concentrations of water (Fig. 6). As with the case of SM-2, poly(SM-2-*co*-MMA) in absolute acetonitrile exhibited a vibronically-structured photoabsorption band in the range of 300 nm to 400 nm and a feeble and vibronically-structured fluorescence band (*λ*^fl^_max_ = *ca.* 420 nm) in the range of 400 nm to 500 nm originating from the anthracene skeleton in the PET active state. The photoabsorption spectra showed unnoticeable changes with the increase in the water content in the acetonitrile solutions. In contrast, the fluorescence intensity of the monomer emission band originating from the anthracene fluorophore increased almost linearly with the increase in the water content in the low water content region below *ca.* 1.0 wt% in the acetonitrile solutions due to the formation of the PET inactive species poly(SM-2-*co*-MMA)a by the addition of water molecules (Fig. 1d), while the fluorescence intensity leveled off when the water content reached 1.0 wt% as with the cases of SM-2 (Fig. 5c). One can see that the acetonitrile solution of poly(SM-2-*co*-MMA) without the addition of water did not show any visual fluorescence emission but exhibited the blue fluorescence emission originating from the anthracene fluorophore upon the addition of water (Fig. 3b). Indeed, the plot of the fluorescence peak intensity at around 420 nm *versus* the water fraction in the low water content region below 1.0 wt% showed that the calibration curve had a good linearity with the *m*_s_ value of 50, intercept value of 2.61, and *R*^2^ value of 0.994 [*F* = 50.4[H_2_O] + 2.61 (*R*^2^ = 0.994, [H_2_O] = 0.0379–0.40 wt%)] (Fig. 5d). However, the *m*_s_ value (50) for poly(SM-2-*co*-MMA) was much smaller than that (355) of SM-2 (Table 1). Based on the calibration curve, the DLs and QLs of poly(SM-2-*co*-MMA) for water in acetonitrile were estimated to be 0.066 and 0.2 wt%, respectively, which were inferior to those (0.009 and 0.028 wt%) of SM-2. The deterioration of the DL and QL values of poly(SM-2-*co*-MMA) compared with SM-2 may be attributed to the dynamic motion of the main chain, to which the anthracene skeleton was directly attached, and hydrophobic environment of the polymer chain, which can inhibit the interaction of the SM-2 moiety with water molecules, leading to the non-radiative decay of the photoexcited anthracene fluorophore. In fact, in acetonitrile with 1.0 wt% water content, the *Φ*_fl_ (5%) of poly(SM-2-*co*-MMA was lower than that (12%) of SM-2. Nevertheless, it was found that the copolymer poly(SM-2-*co*-MMA) composed of SM-2 and MMA can act as a PET-type fluorescent polymeric sensor for the detection and quantification of a trace amount of water in solvents.

**Fig. 7:**
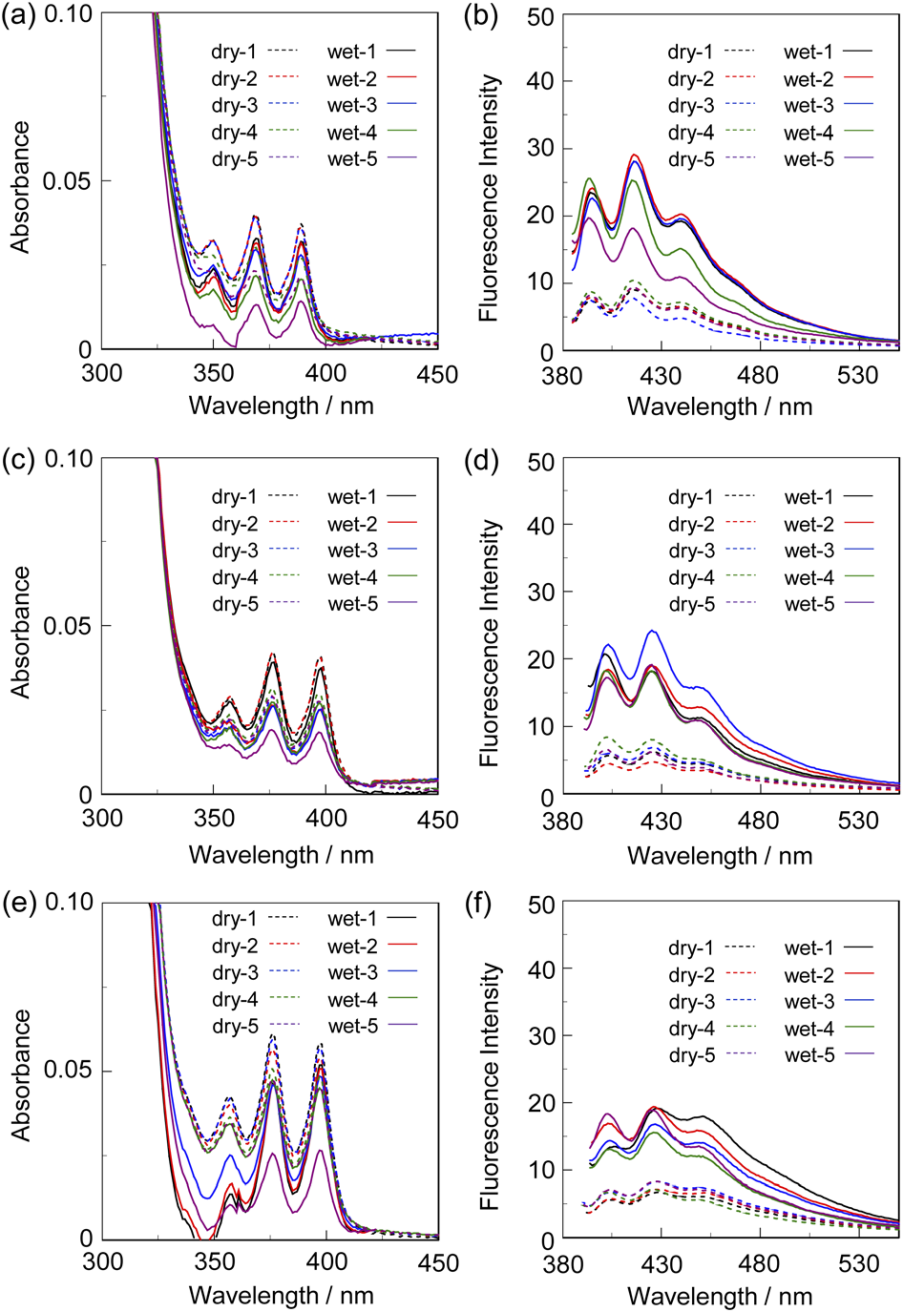
(a) Photoabsorption and (b) fluorescence spectra (*λ*^ex^ = 368 nm) of 15 wt% OF-2-doped PMMA film before (in dry process) and after (in wet process) exposure to moisture. (c) Photoabsorption and (d) fluorescence spectra (*λ*^ex^ = 375 nm) of 15 wt% SM-2-doped PMMA film before (in dry process) and after (in wet process) exposure to moisture. (e) Photoabsorption and (f) fluorescence spectra (*λ*^ex^ = 375 nm) of poly(SM-2-*co*-MMA) film before (in dry process) and after (in wet process) exposure to moisture. For all the photoabsorption spectra, baseline-correction was made to be the same absorbance at 420 nm.

**Fig. 8:**
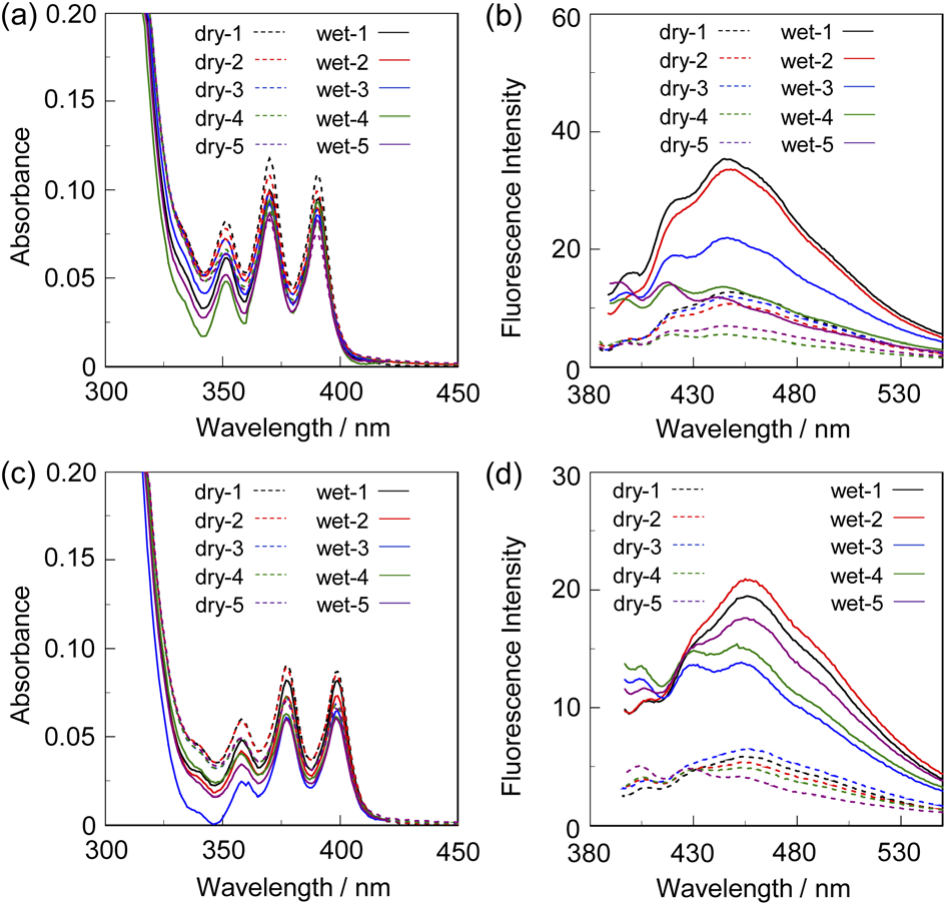
(a) Photoabsorption and (b) fluorescence spectra (*λ*^ex^ = 368 nm) of 50 wt% OF-2-doped PMMA film before (in dry process) and after (in wet process) exposure to moisture. (c) Photoabsorption and (d) fluorescence spectra (*λ*^ex^ = 377 nm) of 50 wt% SM-2-doped PMMA film before (in dry process) and after (in wet process) exposure to moisture. For all the photoabsorption spectra, baseline-correction was made to be the same absorbance at 420 nm.

**Fig. 9:**
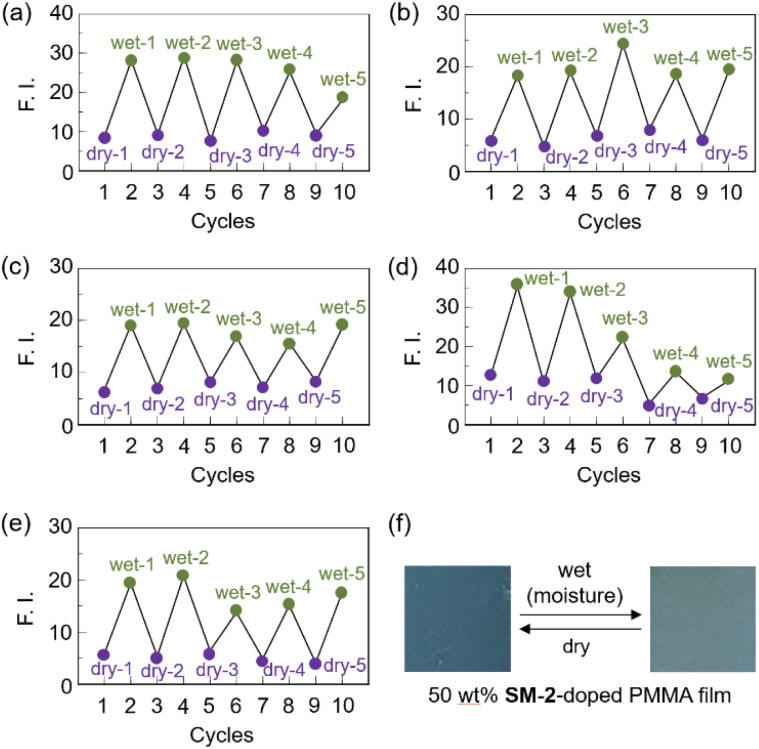
Reversible switching of fluorescence intensity at around 415–430 nm of (a) 15 wt% OF-2-doped PMMA film, (b) 15 wt% SM-2-doped PMMA film, and (c) poly(SM-2-*co*-MMA) film, and at around 450 nm of (d) 50 wt% OF-2-doped PMMA film and (e) 50 wt% SM-2-doped PMMA film during dry–wet process. (f) Photographs (under 365 nm irradiation) of 50 wt% SM-2-doped PMMA film before and after exposure to moisture.

**Fig. 10:**
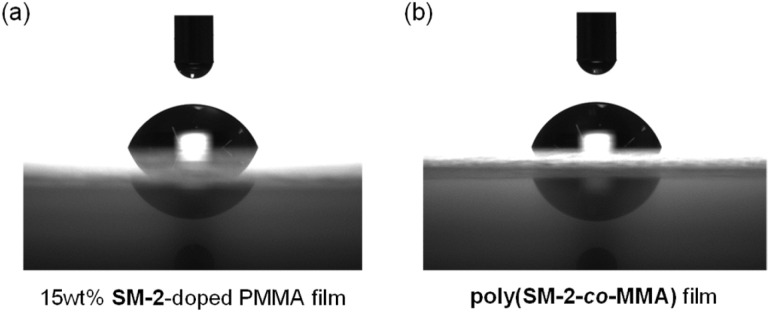
(a) Water contact angle images of (a) 15 wt% SM-2-doped PMMA film and (b) poly(SM-2-*co*-MMA) film.

Next, to evaluate the possibility for the PET-type fluorescent sensor to function in polymer matrices for visualization and detection of water, we prepared spin-coated poly(SM-2-*co*-MMA) films on glass substrates, and photoabsorption and fluorescence spectra of the spin-coated poly(SM-2-*co*-MMA) films before and after exposure to moisture were repeatedly measured several times. In addition, PMMA films doped with OF-2 or SM-2 at 15 wt% as well as 50 wt% were prepared on glass substrates by spin-coating process for comparison with the spin-coated poly(SM-2-*co*-MMA) films, which contained *ca.* 15 wt% SM-2 unit (Fig. 7 and 8). The as-prepared 15 wt% and 50 wt% OF-2- or SM-2-doped PMMA films as well as the poly(SM-2-*co*-MMA) films (in dry process) showed a vibronically-structured photoabsorption band in the range of 300 nm to 400 nm originating from the anthracene skeleton (Fig. 7a, c, e and 8a, c). For the corresponding fluorescence spectra in dry process, the 15 wt% OF-2- or SM-2-doped PMMA films and the poly(SM-2-*co*-MMA) films exhibited a feeble and vibronically-structured fluorescence band with a *λ*^fl^_max_ at around 415–430 nm in the range of 400 nm to 500 nm, which is attributed to the monomer emission originating from the anthracene fluorophore in the PET active state (Fig. 7b, d and f), but the 50 wt% OF-2- or SM-2-doped PMMA films showed a broad and feeble fluorescence band with a *λ*^fl^_max_ at around 450 nm in the range of 400 nm to 600 nm attributable to the excimer emission originating from the anthracene fluorophore in the PET active aggregate state (Fig. 8b and d). When all the OF-2- or SM-2-doped PMMA films and the poly(SM-2-*co*-MMA) films were exposed to moisture (in wet process), the photoabsorption spectral shape did not undergo appreciable changes, although a slight change in the absorbance was observed due to the disturbance of the baselines in the photoabsorption spectra (Fig. 7a, c, e and 8a, c). For the 15 wt% OF-2- or SM-2-doped PMMA films and the poly(SM-2-*co*-MMA) films, the corresponding fluorescence spectra in wet process showed the enhancement of the vibronically-structured monomer emission band originating from the anthracene fluorophore in the PET inactive state (Fig. 7b, d and f). On the other hand, the 50 wt% OF-2- or SM-2-doped PMMA films showed an appearance of the monomer emission band with a *λ*^fl^_max_ at around 415–430 nm and the enhancement of the excimer emission band with a *λ*^fl^_max_ at around 450 nm, that is, the enhancement of the broad fluorescence band originating from the anthracene fluorophore in the range of 400 nm to 600 nm arising from the PET inactive state upon exposure to moisture (Fig. 8b and d). It is worth noting here that when all the OF-2- or SM-2-doped PMMA films and the poly(SM-2-*co*-MMA) films after exposure to moisture were dried in the atmosphere, the photoabsorption and fluorescence spectra recovered the original spectral shapes before exposure to moisture. Actually, one can see that the 15 wt% SM-2-doped PMMA films and the poly(SM-2-*co*-MMA) films initially exhibited visually imperceptible blue emission in the PET active state but the visual blue monomer emission in the PET inactive state upon exposure to moisture (Fig. 3c and d). Meanwhile, the 50 wt% SM-2-doped PMMA films showed feeble green excimer emission in the PET active state before exposure to moisture but the bluish green monomer and excimer emissions in the PET inactive state upon exposure to moisture (Fig. 9f). Therefore, for all the OF-2- or SM-2-doped PMMA films and the poly(SM-2-*co*-MMA) films, the reversibility of the fluorescence intensity at the *λ*^fl^_max_ in the dry–wet process was investigated (Fig. 9a–e). It was found that the dry–wet cycles of the 15 wt% OF-2- or SM-2-doped PMMA films and the poly(SM-2-*co*-MMA) films showed a good reversible switching of the fluorescent intensity even in the five times dry–wet process (Fig. 9a–c). However, the 50 wt% OF-2- or SM-2-doped PMMA films showed that the fluorescence intensity in the wet process was attenuated from the third time onward (Fig. 9d and e). The poor reversibility of the fluorescence intensity of the 50 wt% OF-2- or SM-2-doped PMMA films may be attributed to destruction of the films during the dry–wet process and/or promotion of aggregate formation of OF-2 and SM-2 after dry process. We also measured the water contact angles on the polymer film surfaces to investigate the hydrophilicity of the 15 wt% SM-2-doped PMMA films and the poly(SM-2-*co*-MMA) films (Fig. 10). The water contact angles on the polymer film surfaces were 68.3° and 68.2° for the 15 wt% SM-2-doped PMMA films and the poly(SM-2-*co*-MMA) films, respectively, clearly indicating that the hydrophilicity was similar to each other. Thus, the fact provides the evidence that the both films shows similar reversible switching in the fluorescent intensity in the dry–wet process. Consequently, this work demonstrated that PET-type fluorescent polymer films based on a fluorescence enhancement system produce a satisfactory reversible fluorescence off–on switching between the PET active state and the PET inactive state during the dry–wet process, and thus are one of the most promising and convenient functional dye materials to enable the visualization and detection of moisture and water droplets.

## Conclusions

We have designed and developed the PET-type fluorescent monomer SM-2 composed of methyl methacrylate-substituted anthracene fluorophore-AminoMeCNPhenylBPin and the copolymer poly(SM-2-*co*-MMA) composed of SM-2 and MMA, as fluorescent sensors for visualization, detection, and quantification of a trace amount of water. It was found that both SM-2 and poly(SM-2-*co*-MMA) exhibited enhancement of the fluorescence emission with the increase in water content in various solvents (less polar, polar, protic, and aprotic solvents) due to the formation of the PET inactive (fluorescent) species SM-2a and poly(SM-2-*co*-MMA)a, respectively, by the interaction with water molecules. The detection limit of poly(SM-2-*co*-MMA) for water in acetonitrile was 0.066 wt%, indicating that poly(SM-2-*co*-MMA) can act as a PET-type fluorescent polymeric sensor for a trace amount of water in solvents, although it was inferior to that (0.009 wt%) of SM-2. Moreover, we have achieved the preparation of the spin-coated poly(SM-2-*co*-MMA) films as well as 15 wt% SM-2-doped PMMA films and demonstrated that both the polymer films produced a satisfactory reversible fluorescence off–on switching between the PET active state under a drying process and the PET inactive state upon exposure to moisture. Consequently, this work proposes that PET-type fluorescent polymer films are one of the most promising and convenient functional dye materials to enable the visualization and detection of moisture and water droplets.

## Experimental

### General

Melting points were measured with an AS ONE ATM-02. IR spectra were recorded on a SHIMADZU IRTracer-100 spectrometer by the ATR method. ^1^H and ^13^C NMR spectra were recorded on a Varian-500 FT NMR spectrometer. High-resolution mass spectral data were acquired by APCI on a Thermo Fisher Scientific LTQ Orbitrap XL. Photoabsorption spectra were observed with a SHIMADZU UV-3600 plus. Fluorescence spectra were measured with a Hitachi F-4500 spectrophotometer. The fluorescence quantum yields were determined by a Hamamatsu C9920-01 equipped with a CCD using a calibrated integrating sphere system. The addition of water to 1,4-dioxane, THF, acetonitrile, or ethanol solutions containing SM-2 or acetonitrile solutions containing poly(SM-2-*co*-MMA) was made in terms of weight percent (wt%). The determination of water in solvents was done with MKC-610 and MKA-610 Karl Fischer moisture titrators (Kyoto Electronics Manufacturing Co., Ltd) based on Karl Fischer coulometric titration for below 1.0 wt% and volumetric titration for 1.0–10 wt%. Polymer number-average molecular weights (*M*_*n*_) and molecular weight distributions (*M*_w_/*M*_*n*_) were determined by size exclusion chromatography (SEC) at 40 °C using a SHIMADZU Prominence-i LC-2030 plus with a guard column (LF-G, Shodex), two series-connected columns (LF-804, Shodex), a UV detector, and a differential refractive index detector (RID-20A). THF was used as the eluent, and poly(methyl methacrylate) (PMMA) standards were used to calibrate the SEC system. Static water contact angles were measured at five different positions on a substrate by the sessile drop technique using a Kyowa Interface Science DMo-602 contact angle meter.

### Synthesis

#### (10-(((5-Cyano-2-(4,4,5,5-tetramethyl-1,3,2-dioxaborolan-2-yl)benzyl)(methyl)amino)methyl)anthracen-9-yl)methyl methacrylate (SM-2)

A solution of SM-1 (0.100 g, 0.203 mmol), 4-dimethylaminopyridine (0.003 g, 0.025 mmol), and triethylamine (0.17 mL, 1.21 mmol) in dry THF (15 mL) was stirred for 0.5 h at 0 °C under nitrogen atmosphere, and then, methacryloyl chloride was added slowly to the solution. After stirring for 18 h at room temperature, the reaction mixture was concentrated. The residue was chromatographed on alumina (methanol : dichloromethane = 1 : 100 as eluent) to give SM-2 (0.064 g, yield 56%) as a light yellow solid; m.p. 137–139 °C; FT-IR (ATR): *ν̃* = 2978, 2230, 1713, 1449, 1344, 1315, 1271, 1142 cm^−1^; ^1^H NMR (500 MHz, acetone-*d*_6_): 1.29 (s, 12H), 1.87 (s, 3H), 2.32 (s, 3H), 4.04 (s, 2H), 4.51 (s, 2H), 5.56 (s, 1H), 5.97 (s, 1H), 6.24 (s, 2H), 7.50–7.64 (m, 5H), 7.73 (s, 1H), 7.87 (d, *J* = 7.5 Hz, 1H), 8.46 (d, *J* = 8.7 Hz, 2H), 8.51 (d, *J* = 8.8 Hz, 2H) ppm; ^13^C NMR (125 MHz, CDCl_3_): *δ* = 18.49, 25.13, 42.97, 52.68, 59.45, 60.81, 84.11, 113.64, 119.18, 124.66, 125.54, 125.79, 126.18, 127.21, 129.70, 130.88, 131.28, 132.02, 132.16, 135.57, 136.24, 146.43, 167.72 ppm (two aromatic carbon signals were not observed owing to overlapping resonances); HRMS (APCI): *m*/*z* (%): [M^+^˙] calcd for C_35_H_37_N_2_O_4_B, 560.28409; found 560.28491.

### Preparation of poly(SM-2-*co*-MMA)

A solution of SM-2 (0.023 g, 0.042 mmol), methyl methacrylate (0.100 mL, 0.949 mmol), and azobis(isobutyronitrile) (0.811 mg, 0.005 mmol) in toluene (0.77 mL) was degassed with four freeze–pump–thaw cycles, and then, the solution was stirred for 18 h at 70 °C under nitrogen atmosphere. The reaction mixture was concentrated, and the resulting residue was dissolved in dichloromethane. The dichloromethane solution was poured into *n*-hexane and the resulting precipitate was collected to give poly(SM-2-*co*-MMA) (0.020 g, yield 17%) as a white solid; m.p. 160–200 °C; FT-IR (ATR): *ν̃* = 2991, 2949, 2231, 1728, 1481, 1449, 1387, 1348, 1267, 1240, 1190, 1146 cm^−1^; ^1^H NMR (500 MHz, CDCl_3_): 0.82 (br, C–CH̲_3_ for MMA), 0.82 (br, C–CH̲_3_ for MMA in SM-2 unit), 1.31 (s, CH̲_3_ for BPin), 1.81 (br, CH̲_2_ for MMA and MMA in SM-2 unit), 2.30 (s, N–CH̲_3_ for SM-2 unit), 3.60 (br, O–CH̲_3_ for MMA), 3.96 (s, N–CH̲_2_-Ph for SM-2 unit), 4.50 (s, N–CH̲_2_–An for SM-2 unit), 6.05 (br, O–CH̲_2_–An for SM-2 unit), 7.45–7.60 (m, 4CH̲ at 2,3,6,7-positions on An and CH̲ at 6-position on Ph for SM-2 unit), 7.63 (br, CH̲ at 4- position on Ph for SM-2 unit), 7.83 (br, CH̲ at 3- position on Ph for SM-2 unit), 8.23–8.40 (m, 4CH̲ at 1,4,5,8-positions on An for SM-2 unit) ppm, the molar ratio (*y*/*x*) of MMA unit (*y*) and SM-2 unit (*x*) and the weight percentage (wt%) of SM-2 in the obtained poly(SM-2-*co*-MMA) was determined to be *ca.* 40 and *ca.* 15 wt%, respectively, from the ^1^H NMR spectrum; SEC *M*_*n*_ = 18 900, *M*_w_/*M*_*n*_ = 2.08; UV-Vis *λ*^abs^ = 339, 356, 375, 395 nm, PL *λ*^fl^ = 401, 422, 447, 480 nm (in acetonitrile).

### Preparation of poly(SM-2-*co*-MMA) film

A solution of poly(SM-2-*co*-MMA) (8.0 mg) in toluene (0.4 mL) was stirred for 3 h at room temperature, while poly(SM-2-*co*-MMA) has dissolved quickly. To prepare a polymer film, 150 μL of a poly(SM-2-*co*-MMA) solution was spin-coated (1000 rpm for 30 s) on a glass substrate (MIKASA MS-A-100 Opticoat Spincoater). The spin-coated films were dried in air. The resulting poly(SM-2-*co*-MMA) films were exposed to moisture for 60 s using a humidifier.

### Preparation of 15 wt% and 50 wt% OF-2- or SM-2-doped PMMA films

A solution of PMMA (8.5 mg and 5.0 mg for 15 wt% and 50 wt%, respectively) in toluene (0.5 mL) was stirred for several hours at 60–70 °C until PMMA has dissolved, and then, OF-2 or SM-2 (1.5 mg and 5.0 mg for 15 wt% and 50 wt%, respectively) was added to the solution. To prepare a polymer film, 150 μL of a OF-2-PMMA solution or a SM-2-PMMA solution was spin-coated (1000 rpm for 30 s) on a glass substrate (MIKASA MS-A-100 Opticoat Spincoater). The spin-coated films were dried in air. The resulting 15 wt% and 50 wt% OF-2- or SM-2-doped PMMA films were exposed to moisture for 60 s using a humidifier.

## Conflicts of interest

There are no conflicts to declare.

## Supplementary Material

RA-012-D2RA03894C-s001
