# Supplementary material for: Fluorescent polymer films based on photo-induced electron transfer for visualizing water
Source: RSC Adv. 2022 Sep 9;12(39):25687–96. doi: 10.1039/d2ra03894c (PMC9462076; doi:10.1039/d2ra03894c)
Supplement: RA-012-D2RA03894C-s001 [file RA-012-D2RA03894C-s001.pdf]

Electronic Supplementary Material (ESI) for RSC Advances.
This journal is © The Royal Society of Chemistry 2022

Supplementary Information

Fluorescent polymer films based on photo-induced electron transfer for visualizing water

Saori Miho, Keiichi Imato* and Yousuke Ooyama*

Applied Chemistry Program, Graduate School of Advanced Science and Engineering, Hiroshima University, 1-4-1 Kagamiyama, Higashi-Hiroshima 739-8527, Japan. E-mail: yooyama@hiroshima-u.ac.jp

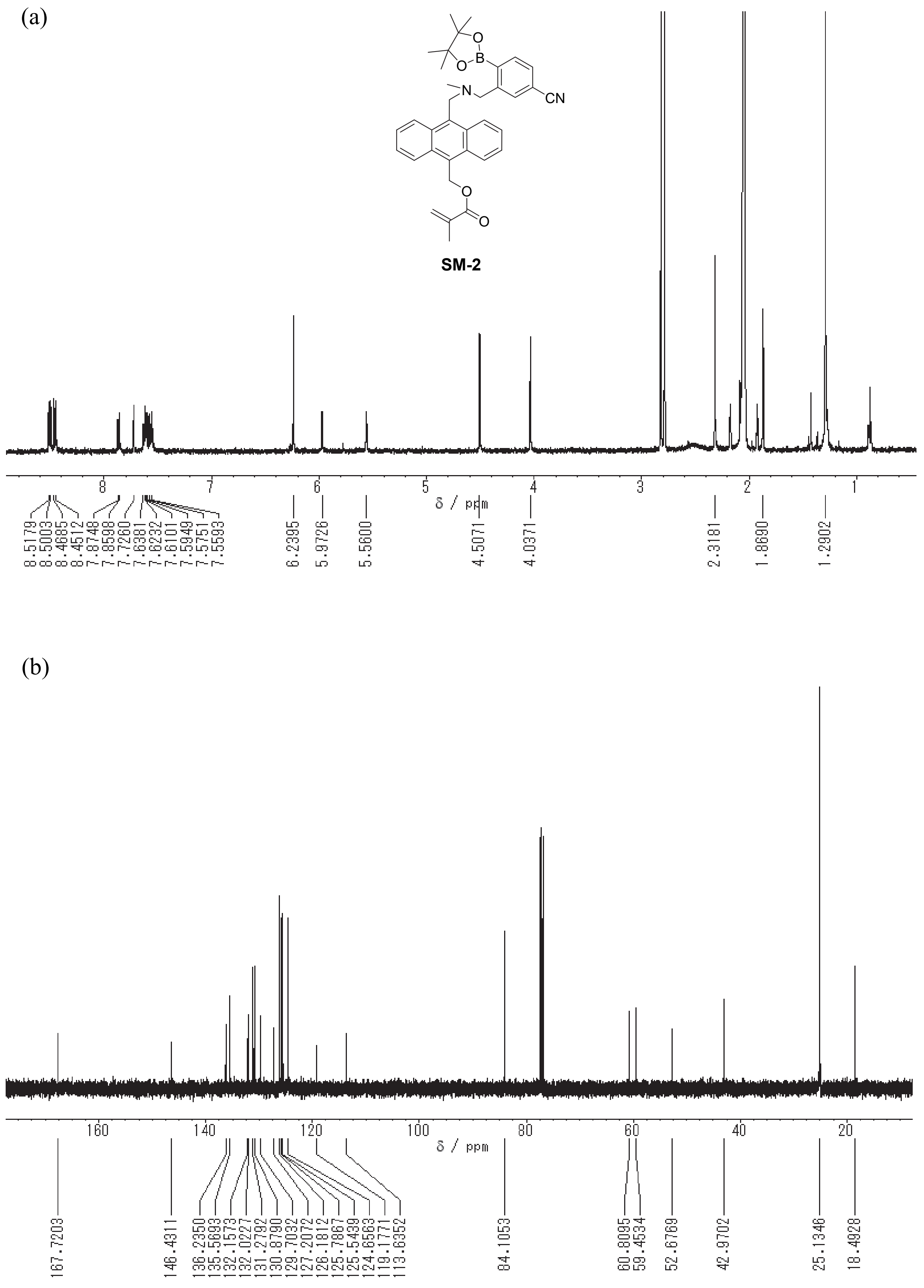

Fig. S1 (a) ^{1}H NMR (500 MHz) spectrum of **SM-2** in acetone-d_6. (b) ^{13}C NMR (125 MHz) spectrum of **SM-2** in $CDCl_3$.

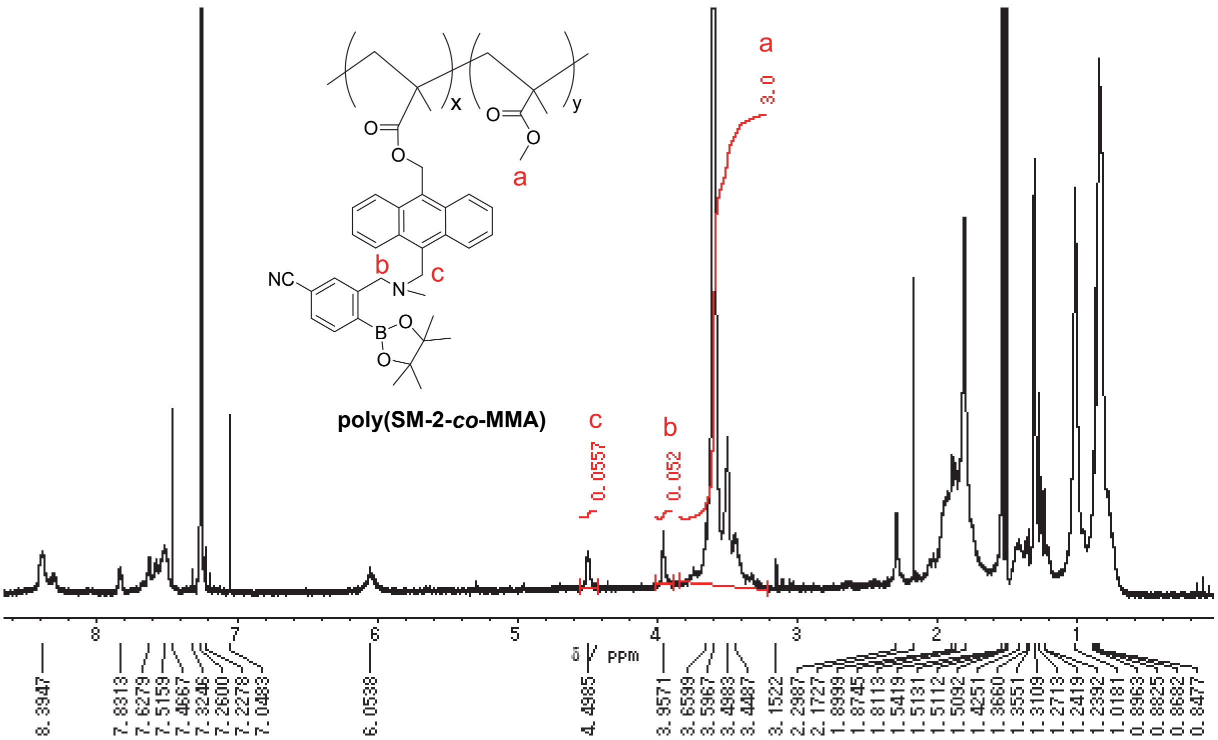

Fig. S2 ^{1}H NMR (500 MHz) spectrum of **poly(SM-2-*co*-MMA)** in $CDCl_3$.

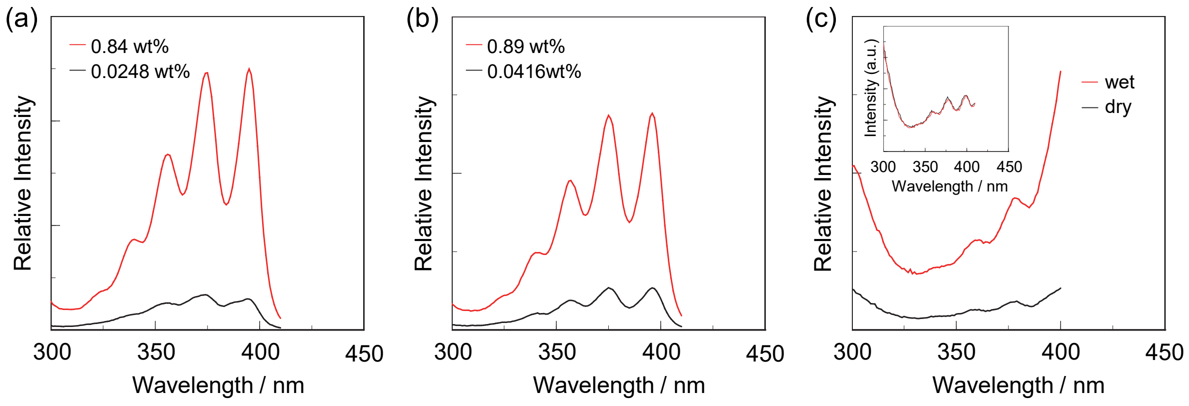

Fig. S3 Photoluminescence excitation spectra (λ^{fl} = ca. 420−430 nm) of (a) **SM-2** (2.0×10^{-5} M) in acetonitrile containing water (0.0248 wt% and 0.84 wt%), (b) **poly(SM-2-*co*-MMA)** in acetonitrile containing water (0.0416 wt% and 0.89 wt%), and (c) **poly(SM-2-*co*-MMA)** film before (in dry process) and after (in wet process) exposure to moisture; the inset in (c) is the normalized photoluminescence excitation spectra (λ^{fl} = 440 nm) of **poly(SM-2-*co*-MMA)** film before (in dry process) and after (in wet process) exposure to moisture.
